# The Association between Active and Passive Smoking and Latent Tuberculosis Infection in Adults and Children in the United States: Results from NHANES

**DOI:** 10.1371/journal.pone.0093137

**Published:** 2014-03-24

**Authors:** Ryan P. Lindsay, Sanghyuk S. Shin, Richard S. Garfein, Melanie L. A. Rusch, Thomas E. Novotny

**Affiliations:** 1 University of California San Francisco, Center for Tobacco Control Research and Education, San Francisco, California, United States of America; 2 University of California Los Angeles, David Geffen School of Medicine, Program in Global Health, Los Angeles, California, United States of America; 3 University of California San Diego, Department of Medicine, Division of Global Public Health, San Diego, California, United States of America; 4 Vancouver Island Health Authority and University of Victoria, School of Public Health and Social Policy, Victoria, British Columbia, Canada; 5 San Diego State University, Graduate School of Public Health, San Diego, California, United States of America; The Catholic University of the Sacred Heart, Rome, Italy

## Abstract

**Background:**

Few studies assessing the relationship between active and passive smoking and tuberculosis have used biomarkers to measure smoke exposure. We sought to determine the association between active and passive smoking and LTBI in a representative sample of US adults and children.

**Methods:**

We used the 1999–2000 US National Health and Nutrition Examination Survey (NHANES) dataset with tuberculin skin test (TST) data to assess the association between cotinine-confirmed smoke exposure and latent tuberculosis infection (LTBI) among adults ages ≥20 years (n = 3598) and children 3–19 years (n = 2943) and estimate the prevalence of smoke exposure among those with LTBI. Weighted multivariate logistic regression was used to measure the associations between active and passive smoking and LTBI.

**Results:**

LTBI prevalence in 1999–2000 among cotinine-confirmed active, passive, and non-smoking adults and children was 6.0%, 5.2%, 3.3% and 0.3%, 1.0%, 1.5%, respectively. This corresponds to approximately 3,556,000 active and 3,379,000 passive smoking adults with LTBI in the US civilian non-institutionalized population in 1999–2000. Controlling for age, gender, socioeconomic status, race, birthplace (US vs. foreign-born), household size, and having ever lived with someone with TB, adult active smokers were significantly more likely to have LTBI than non-smoking adults (AOR = 2.31 95% CI 1.17–4.55). Adult passive smokers also had a greater odds of LTBI compared with non-smokers, but this association did not achieve statistical significance (AOR = 2.00 95% CI 0.87–4.60). Neither active or passive smoking was associated with LTBI among children. Among only the foreign-born adults, both active (AOR = 2.56 (95% CI 1.20–5.45) and passive smoking (AOR = 2.27 95% CI 1.09–4.72) were significantly associated with LTBI.

**Conclusions:**

Active adult smokers and both foreign-born active and passive smokers in the United States are at elevated risk for LTBI. Targeted smoking prevention and cessation programs should be included in comprehensive national and international TB control efforts.

## Introduction

Tuberculosis (TB) remains a substantial global health problem, with an estimated one out of every three people worldwide infected with *Mycobacterium tuberculosis* [1] and 8.6 million incident active cases globally in 2012 [2]. Smoking, though declining in the United States, is still a major cause of morbidity and mortality in the United States and worldwide. Smoking has consistently been shown to be a risk factor for poor TB outcomes including activation of latent TB infection (LTBI) to TB disease, progression of active TB, and TB-related mortality [3], [4], [5]. There is less evidence and agreement on the role that smoking may have on transmission and infection with TB.

While active or former smoking has been associated with increased risk for LTBI [6], [7], [8], [9], [10], [11], [12], including a population-based national sample in the United States [13], fewer studies have investigated the association between passive smoking (secondhand smoke exposure) and LTBI in adults [14] or children [15], [16], [17]. All studies reported a positive association between passive smoking and LTBI, though some did not adjust for confounding variables [16], [17], and one study found no association after adjusting for confounding variables [15]. Among studies that assessed a dose-response relationship between smoke exposure levels, Shin et. al. reported an increased risk of LTBI with increased smoke exposure [14] while Horne et. al. reported a dose-response relationship only among non-Hispanic blacks in the United States but no overall association among adults of all race/ethnicity between past or present smoke exposure and LTBI in a study using the 1999-2000 NHANES dataset [13].

Passive smoke exposure may contribute to the household spread of TB, and contacts of TB patients who smoke have been shown to have an independent and increased risk of LTBI [18]. As children are thought to be more susceptible to the effects of passive smoking on their immature respiratory and immune systems [19], [20], they may be at particular risk of TB infection when exposed to secondhand smoke [21]. The association between passive smoking with LTBI has not yet been investigated on a representative sample in the United States.

In many previous studies on the relationship between passive smoking and LTBI researchers have relied on self-reported smoking status to assess risk [15], [16], [17]; this measurement can be subject to significant underreporting bias. Furthermore, variations in smoking behavior that can affect pathophysiology, such as depth of inhalation, are difficult to capture via self-report [22]. Cotinine, a metabolite of nicotine, may be measured in a variety of body fluids and is considered the gold-standard for assessing nicotine exposure [23]. While self-reported current smoking status is 80–90% concordant with measured serum cotinine levels in national studies in the United States [24], estimating exposure to passive smoke among non-smokers is more difficult [25]. Furthermore, there is less concordance between biologic assessment of smoking status and self-report among adolescents, among whom smoking may be more sporadic and where socially desirable responding may lead to under-reporting of current or passive smoking [22].

We sought to determine the association between active and passive smoking and LTBI in a representative sample of the US population using cotinine verification of smoking exposure among non-smoking adults and children.

## Methods

### Data source

The National Health and Nutrition Examination Survey(NHANES) is administered in 2 year cycles by the Centers for Disease Control and Prevention's (CDC) National Center for Health Statistics (NCHS) and is a nationally representative sample of the US civilian (non-institutionalized) population ≥1 year of age, with oversampling of persons aged ≥60 years and minority racial/ethnic groups [26]. The 1999–2000 NHANES survey involved a home interview followed by a lab interview at the time of physical examination, tuberculin skin test (TST) placement, and biological specimen collection. Biomarkers included serum cotinine. Respondents returned to have TST read 48–72 hours after placement. The 1999–2000 NHANES survey was the most recent publically available survey cycle that included both cotinine and TST data. The unweighted sample size of adults for the home interview was 4880 (response rate  = 76.2%) and for the examination was 4444 (response rate  = 69.4%). For children 1–19 years old, 4612 were interviewed (response rate 88.0%) and 4388 examined (response rate 83.8%). Only children ≥3 years old had TSTs placed. Among adults and children, 97% of those who had a physical examination returned to have their TST read [27].

### Ethics statement

The NHANES study protocol was approved by the NCHS Institutional Review Board (IRB). Since the current analysis used only de-identified data, it was exempt from IRB review.

### TB and smoking exposure classification

After TST placement, induration was measured 48–72 hours later by trained readers. Induration ≥10 mm was considered a positive result for LTBI. For those with evidence of Bacillus Calmette-Guérin(BCG) vaccination (determined by the presence of a scar on the upper arm during the medical examination), induration of ≥15 mm was considered positive. There were 39 adults and 4 children that were BCG-vaccinated and had induration between 10–15 mm that were not considered to have LTBI.

Self-reported current smoking behavior was measured through the home interview followed by the lab interview conducted at the time of the physical examination, when serum sampling is completed. At the home interview adults that had smoked at least 100 cigarettes in their life were asked “Do you currently smoke cigarettes?”. At the lab interview adults and adolescents 12–19 years were asked “During the past 5 days, did you use any product containing nicotine including cigarettes, pipes, cigars, chewing tobacco, snuff, nicotine patches, nicotine gum, or any other product containing nicotine?” and if adolescents indicated they had ever tried smoking they were also asked “During the past 30 days, on how many days did you smoke cigarettes?”.

Participants were classified as non-, passive, or active smokers using information from both the home and lab interviews and the laboratory results using CDC definitions [28] (Table 1). Persons with serum cotinine levels <0.05 ng/mL (0.05 ng/mL being the minimum level of detection (LOD) for the 1999–2000 survey) who did not report smoking prior to their physical examination were classified as non-smokers. Those who reported no smoking but had serum cotinine levels of 0.05–10 ng/mL were classified as passive smokers. Participants with serum cotinine levels >10 ng/mL who reported smoking cigarettes, pipes, or cigars, were considered active smokers. Persons who reported smoking and had serum cotinine levels <10 ng/mL were considered active smokers (likely light or non-daily smokers) [24].Where there were discrepant responses regarding current smoking in between interview questions, or interview questions with regards to current smoking behavior were missing, we then used the above described serum cotinine cutoff points to categorize smoking exposure. Children aged 3–12 years were not interviewed regarding smoking and were assigned to exposure categories using only serum cotinine levels; cotinine levels ≥0.05 ng/mL defined children as passive smokers. Of the 3856 adults and 3206 children who had TST results, 203 adults (unweighted proportion 5.6%) and 253 children (unweighted proportion 7.9%) were missing serum cotinine lab results and were excluded from this analysis. Since cotinine measurements could be influenced by smokeless tobacco products or cessation aids containing nicotine, 55 adults (unweighted proportion 1.5%) and 10 children (unweighted proportion 0.3%) with cotinine lab results that reported using smokeless tobacco products or cessation aids without self-reported smoking were also excluded from this analysis.

**Table 1:** Determination of smoking status among those with complete tuberculin skin test (TST) results.

	Smoking status	Adults (unweighted n = 3598)	Children (unweighted n = 2943)	
		Ages 20+	Ages 12–19	Ages 3–11∧
Active smoking	Self-reported smoker w/cotinine ≥10 ng/mL	849	178	-
	Discrepant self-report w/cotinine ≥10 ng/mL	10	29	-
	Missing self-report w/cotinine ≥10 ng/mL	32	17	-
	Self-reported non-smoker w/cotinine ≥10 ng/mL	47	27	-
	Self-reported smoker w/cotinine <10 ng/mL	86	66	-
	Subtotal	938	317	-
Passive smoking	Self-reported non-smoker w/cotinine ≥ 0.05 ng/mL & <10 ng/mL	1206	817	-
	Discrepant self-report w/cotinine ≥ 0.05 ng/mL & <10 ng/mL	14	42	-
	Missing self-report w/cotinine≥ 0.05 ng/mL & <10 ng/mL (≥0.05 for children 3–11)	3	66	682
	Subtotal	1223	925	682
Non-smoking	Self-reported non-smoker w/cotinine <0.05 ng/mL	1435	586	-
	Discrepant self-report w/cotinine <0.05 ng/mL	2	19	-
	Missing self-report w/cotinine <0.05 ng/mL	0	25	389
	Subtotal	1437	630	389
	Total	3598	1872	1071

∧Ages 3-11 did not receive smoking behavior questions

Covariates of interest included age at time of examination (approximately 4–6 weeks after the home interview), gender, race/ethnicity, country of birth (United States, Mexico, Other foreign-born), poverty income ratio (<1 indicates below the poverty line, adjusted by year), level of education, the number of persons living in the same household, and whether the participant had ever lived with someone with active TB. Frequency of alcohol use among adult participants with complete TST tests and smoking status had a substantial amount of missing data (15.3%); we performed a sensitivity analysis to compare the inclusion of heavy alcohol use (≥5 drinks per day) in the multivariate model for adults.

### Statistical Analysis

Adults ≥20 years of age and children ≥3-19 were analyzed separately due to potential biological differences in respiratory and immune systems. Additionally, adults and children (aged 13–19) received different questionnaires ascertaining smoking behavior. Weighted analyses were used to account for oversampling of the elderly, and minority race/ethnicity subpopulations. To account for non-response for those without a complete TST reading, the two-year Taylor series weights provided by the NCHS for NHANES were adjusted according to Bennett et al. (see online supplement) [27]. Chi-square and t-tests were used to calculate differences between smoking status and covariates and LTBI status for nominal and continuous measures, respectively. Associations between smoking status and LTBI were explored using weighted bivariate analyses and multivariate logistic regression using Stata version 12 (StataCorp. College Station, TX).

## Results

### Sociodemographic correlates of LTBI

Respondents with LTBI were more likely to have been born outside the United States, and have lower household income than those who did not have LTBI (Table 2). Among adults, respondents with LTBI were more likely to be male, of minority race/ethnicity, with less than high school education, living in larger households than LTBI-negative participants, and report having lived with someone with active TB. Among children, age, gender, race/ethnicity, having lived with someone with active TB, and household size did not significantly differ by LTBI status. Overall, there was no difference in LTBI status based on BCG vaccination (p = 0.42 for adults and p = 0.78 for children, Pearson's Chi-square). There was no association between heavy alcohol use and LTBI among adults (OR = 1.17, 95% CI 0.45–3.06). Heavy alcohol use was included in a multivariate model that included n = 2634 adults with alcohol data; the relationship between active and passive smoking and LTBI was strengthened (AOR = 3.41, 95% CI 1.91–6.10 and AOR = 2.39, 95% CI 1.16–4.92, respectively).

**Table 2:** Latent TB Infection among US non-institutionalized adults (aged ≥20 years) and children (aged 3–19 years) by sociodemographic correlates and smoking status, 1999–2000.

	Adults			Children		
	LTBI-Unweighted n = 3317 (weighted %)	LTBI+ Unweighted n = 281 (weighted %)	P (Pearson's Chi-square)	LTBI- Unweighted n = 2885 (weighted %)	LTBI+ unweighted n = 58 (weighted %)	P
Smoking status			0.046			0.387
Non-smoker	1335 (35.8)	102 (24.5)		987 (31.0)	32 (41.3)	
Passive smoker	1135 (33.6)	88 (36.8)		1585 (58.5)	22 (56.2)	
Active smoker	847 (30.7)	91 (38.7)		313 (10.5)	4 (2.5)	
Age (years)			0.068			0.999
Weighted mean	45.3	48.7		11.6	11.6	
Male sex	1499 (46.3)	174 (61.5)	0.010	1505(53.8)	38 (50.8)	0.847
Race/Ethnicity			<0.001			0.108
Non-Hispanic White	1615 (73.2)	37 (30.7)		593 (59.0)	5 (55.5)	
Mexican American	849 (6.0)	140 (17.2)		1214 (11.5)	46 (29.2)	
Non-Hispanic Black	586 (9.7)	65 (19.6)		832 (15.1)	5 (07.9)	
Other Hispanic/Multiracial/Other	267 (11.1)	39(32.6)		246 (14.4)	2 (07.4)	
Birthplace			<0.001			<0.001
US	2550 (85.3)	114 (40.1)		2512 (92.2)	22 (30.1)	
Mexico	456 (3.3)	112 (15.2)		277 (2.1)	30 (19.0)	
Other country	308 (11.3)	55 (44.7)		95 (5.8)	6 (50.9)	
Ever lived with someone with active TB	136 (3.3)	35 (12.9)	<0.001	40 (0.8)	4 (2.6)	0.072
BCG scar	466 (12.9)	35 (17.5)	0.418	42(0.8)	0(0.0)	0.777
Education			<0.001			-
< High School graduate	1182 (22.9)	185 (49.0)		-	-	
High school graduate/GED or more	2115 (77.1)	95 (51.1)		-	-	
Poverty Income Ratio<1	566 (15.1)	68 (26.9)	0.029	979 (27.7)	31 (87.1)	<0.001
Household size (persons)			<0.001			0.618
Weighted mean	3.0	3.6		4.4	4.7	

### Active and passive smoking and LTBI

Adults with cotinine-confirmed active smoking status were almost two times as likely to have LTBI (OR = 1.85, 95% CI 1.09–3.14) compared to non-smokers (Table 3). After adjusting for age, gender, poverty income ratio, race, education, birthplace, household size, and having ever lived with somebody with TB, the odds ratio (OR) for the association between active smoking and LTBI among adults was 2.31 (95% CI 1.17–4.55) (Table 4). The adjusted OR for LTBI among cotinine-confirmed passive smoking among adults was 2.00 (95% CI 0.87–4.60). Neither active or passive smoking was associated with LTBI in multivariate analyses for children.

**Table 3:** Bivariate logistic regression of smoking status and other sociodemographic correlates, and LTBI, United States, 1999–2000.

	Adults (≥20) n = 3843	Children (3–19) n = 2943
	OR (95% CI)	OR (95% CI)
Smoking status (Ref = non-smokers)		
Passive smokers	1.60 (0.92–2.80)	0.72 (0.18–2.86)
Active smokers	1.85 (1.09–3.14)	0.18 (0.04–0.89)
Age (5-year intervals for adults, 2-year intervals for children)	1.00(1.00–1.02)	1.00 (0.93–1.07)
Male sex	1.85(1.18–2.91)	1.12 (0.31–4.16)
Race/ethnicity (ref = non-Hispanic white)		
Mexican American	6.79 (3.66–12.62)	2.70 (0.74–9.85)
Non-Hispanic Black	4.81 (2.59–8.93)	0.56 (0.12–2.66)
Other Hispanic/multiracial/Other	7.01 (3.57–13.77)	0.54 (0.07–4.11)
Education (ref = Beyond High School)		
< High school graduate	3.23 (2.16–4.83)	-
Poverty income ratio <1	2.07(1.08–3.98)	17.68 (5.77–54.16)
Birthplace (Ref = born in US)		
Mexican-born	9.64(6.70–13.88)	27.79 (7.99–96.66)
Foreign-born	8.41(4.98–14.21)	27.07 (5.13–142.64)
Size of household (unit = 1 person)	1.27(1.15–1.39)	1.17 (0.60–2.28)
Ever lived w/someone w/active TB	4.40(2.29–8.44)	3.39 (0.81–14.21)

**Table 4:** Multivariable analysis* of smoking status and LTBIrisks by age and birthplace,United States, 1999-2000.

	All Adults (n = 3092)	All Children (n = 2563)	U.S. born adults (n = 2319)	U.S. born children (n = 2222)	Foreign born adults (n = 773)	Foreign born children (n = 302)
	Adjusted OR	Adjusted OR	Adjusted OR	Adjusted OR	Adjusted OR	Adjusted OR
Smoking Status (Ref = non-smokers)						
Passive smokers	2.00(0.87–4.60)	0.40 (0.07–2.24)	1.46 (0.59–3.63)	0.07 (0.01–0.42)	2.27 (1.09–4.72)	1.31 (0.40–4.23)
Active smokers	2.31(1.17–4.55)	0.13 (0.01–1.44)	1.73 (0.80–3.76)	0.18 (0.05–0.66)	2.56 (1.20–5.45)	0.06 (0.00–0.75)

*All models for adultswere adjusted for age, gender, poverty income ratio, race, education,household size, and having ever lived with someone withTB (except

when stratified by birthplace).Models for children were adjusted using the same variables as for adults except for education.

The strongest correlate of LTBI for both adults and children in this sample was foreign-born status (Table 3).Therefore, we tested the association of smoking and LTBI among adults and children stratifying by place of birth (Table 4). Among foreign-born adults, the association between active and passive smoking and LTBI was stronger than for US-born adults. US and foreign-born children who were active smokers as well as US born passive smoking children were paradoxically less likely to have LTBI compared with non-smokers.

### Prevalence of LTBI

The prevalence of LTBI overall was 4.8% (95% CI 3.4%–6.1%) for adults and 1.1% (95% CI 0.3%–1.9%) for children aged 3–19 (Table 5). The prevalence of LTBI was 6.0%, 5.2%, and 3.3% for adult active smokers, passive smokers, and non-smokers, respectively. The prevalence of LTBI among children ages 3–19 was 0.3% for active smokers and 1.1% for passive smokers and 1.5% for non-smokers. The prevalence of LTBI among US born adults was 3.01%, 2.44%, and 1.50% for active smokers, passive smokers and non-smokers, compared to 24.0%, 18.3%, and 11.2% among foreign-born adults, respectively. In the same respective smoking categories, US born children had an LTBI prevalence of 0.3%, 0.1%, and 0.9% compared to 0.5%, 12.5%, and 7.1% among foreign-born children.

**Table 5:** Estimated US prevalence of LTBI according to smoking characteristics by age and birthplace, 1999–2000.

	Adults			Children		
Characteristics	Weighted LTBI prevalence	Population with characteristic	Estimated no. with LTBI	Weighted LTBI prevalence	Population with characteristic	Estimated no. with LTBI
	% (95% CI)	No. x 1,000	No. x 1,000 (95% CI)	% (95% CI)	No. x 1,000	No. x 1,000 (95% CI)
All participants	4.76 (3.44–6.09)	192748	9184 (6637–11730)	1.13 (0.33–1.94)	69500	789 (226–1352)
Smoking						
Overall						
Non-smokers	3.31 (2.31,4.72)	67895	2248 (1569–3208)	1.51 (0.64–3.50)	21638	326 (139–757)
Passive	5.20 (3.31–8.06)	65043	3379 (2154–5242)	1.01 (0.38–3.10)	40624	443 (154–1260)
Active	5.95 (4.20,8.36)	59811	3556 (2509–5001)	0.27 (0.06–1.19)	7238	20 (5–86)
U.S. Born						
Non-smokers	1.50 (0.94–2.40)	55142	827 (516–1321)	0.88 (0.25–3.08)	19469	171 (48–599)
Passive	2.44 (1.49–3.96)	53732	1310 (801–2130)	0.13 (0.04–0.41)	37477	50 (16–153)
Active	3.01 (1.97–4.56)	51464	1549 (1016–2348)	0.26 (0.05–1.25)	6609	17 (3–83)
Foreign Born						
Non-smokers	11.16 (6.92–17.51)	12741	1422 (882–2231)	7.14 (2.62–18.04)	2171	155 (57–392)
Passive	18.34 (11.24–28.49)	11290	2071 (1269–3216)	12.52 (5.16–27.32)	3143	393 (162–859)
Active	23.98 (16.48–33.52)	8380	2009 (1381–2809)	0.47 (0.05–4.24)	630	3 (0–27)

### Prevalence of active and passive smoking

After weighting all data and using serum cotinine to confirm smoking status, 28.2% (95% CI 24.0%–32.5%) of adults and 21.3% (95% CI 16.8%–25.8%) among those 12–19 years of age were active smokers in the United States in 1999–2000; 36.5% (95% CI 33.9%–39.1%) of adults and 58.5% (95% CI 54.5%–62.4%) of children aged 3–19 were passive smokers; and 35.2% (95% CI 30.1%–40.3%) of adults and 31.1% (95% CI 25.6%–36.6%) of children aged 3–19 were non-smokers.

## Discussion

This study of the non-institutionalized US population confirms the positive association between cotinine-confirmed active smoking and LTBI among adult participants. In addition, after stratification by foreign-born status, the risk of LTBI due to active smoking was even greater among adults born outside of the United States and there was no association between active smoking and LTBI among US-born adults. Passive smoking had a marginally significant association with LTBI among US-born adults and a significant association with LTBI among the foreign-born adults. The effect of smoking and smoke exposure may be particularly important where there is a higher prevalence of *Mycobacterium tuberculosis* circulating in the community, which is the case for many nations from which the foreign born population in the United States emigrate. In fact, den Boon et al. found that passive smoking was only associated with LTBI among those living with an active TB patient and was not associated among those not living with an active TB patient [15]. Therefore, active and passive smoking in immigrants' home countries along with higher prevalence of *M. tuberculosis* may be an important determinant of the US TB burden.

We did not find that passive smoking was associated with increased risk for LTBI among children in contrast with other published studies on LTBI [15], [16], [17], [29] and active TB [30], [31] among children. This lack of association could simply be due to the small number of LTBI cases among children in the NHANES 1999–2000. However, all other studies were conducted among contacts of TB patients or in high TB incidence areas and used self-reported smoking measures, which could affect risk estimates. Two of these studies reported an association between passive smoking and TB infection among children living in the home of a TB patient [15], [17]. Du Preez et al. reported a dose-response relationship between passive smoke exposure and LTBI in a high TB-incidence area [29]. In the only other study of passive smoking and LTBI among children conducted in the United States, Kuemmerer did not exclude active smokers or control for known confounding factors; in addition LTBI prevalence and smoking prevalence were much higher at the time of his study (1963) than in 1999–2000, and the control group would thus have experienced more extensive passive smoke exposure. We therefore suspect that our differing results may be attributable to the low prevalence of LTBI in the US population (especially among children), differences in measurement of passive smoke exposure, and the difference in passive smoke exposure in the US population of 1999–2000 compared with other study populations. More research is needed among adults and children using cotinine-confirmed measures of passive smoking in settings with diverse TB exposures.

There is increasing mechanistic evidence providing biological plausibility that smoking increases the likelihood of TB infection [32], [33], [34]. Smoke exposure reduces normal ciliary function of pathogen clearance from the lung and bronchial pathways [35]. Alveolar macrophages in smokers are less able to respond to harmful bacteria in the lung [36]. In addition, systemic immune response is compromised in smokers [32]. Finally, smoke exposure results in more coughing [37], [38] that may facilitate aerosolizing *M. Tuberculosis* from the lungs of infected smokers, which could lead to increased transmission.

Since smoking has been associated with poor adherence to treatment for both active TB and LTBI [39], [40],_ENREF_37 and given our approximation of 6.9 million adults who were either active or passive smokers with LTBI in the US in 1999–2000 (4.1 million of whom are foreign-born), current efforts to treat LTBI in the US may benefit with special attention to tobacco control policies and smoking cessation interventions among adults generally and the foreign-born with LTBI. With recent changes in treatment guidelines for LTBI [29], more research is needed to determine if there is an association between LTBI treatment failure under new guidelines and active and passive smoking exposure.

### Limitations

We used a cross-sectional design for this study; thus, the temporal relationship between smoking and the timing of *M. tuberculosis* infection could not be determined. However, cohort studies needed to identify onset of smoking, onset of LTBI, and length and intensity of exposure as antecedents to LTBI are impractical given the large sample size and long follow-up period necessary. Furthermore, a limitation of using serum cotinine to assess the association between smoking and LTBI is that its half-life is 18–20 hours [41]. Thus, serum cotinine is not a perfect indicator of exposure to either active or passive smoke, as persons who had stopped smoking a few days prior to the examination would not have accurate cotinine measurements.

We used a conservative cutoff point of 10 ng/mL to distinguish active smokers from non-smokers; however, some researchers have recommended even lower cutoff points, ranging from 1–6 ng/mL depending on age and race [42] to ascertain current smoking status. Though our cutoff point of 10 ng/mL may have misclassified some light or non-daily smokers as passive smokers potentially resulting in an overestimation of the association between passive smoking and LTBI, by triangulating cotinine-levels with interview questions, any misclassification of smoking status using the cutoff point of 10 ng/mL would have been more likely among those with discrepant or missing interview responses. Discrepant or missing interview responses were few among adults and more common among adolescents.

A challenge of investigating the association between smoking and LTBI in the US population is the low prevalence of LTBI. This may have affected our LTBI risk estimates among children, and, as a result, the confidence limits around our risk estimates for LTBI were quite wide. While the prevalence estimates are more than a decade old, measuring the association between active and passive smoking and LTBI at the US population level may become increasing challenging as the prevalence of smoking, passive smoke exposure and LTBI has decreased since 1999–2000. In addition, the TST test is unable to identify active TB cases from LTBI possibly resulting in an overestimation of LTBI prevalence, though active TB is rare in the United States. Further discussion of the limitations of the TST test and confounders of LTBI in the NHANES study can be found elsewhere [27]. While TST may have overestimated LTBI in the population, we had a more strict definition of LTBI using a criterion of ≥15 mm skin induration (instead of 10 mm) for infection for those who had been vaccinated with BCG. Though we controlled for many potential confounders, it is possible that the apparent associations between smoking and LTBI in our study remain influenced by residual confounding from un- or inadequately measured covariates.

## Conclusion

While the effects of active and passive smoking on LTBI in the United States across age and country of birth are not consistent in the 1999–2000 NHANES, active smoking among all adults, and active and passive smoking among the foreign-born adults, were strongly associated with LTBI. Further research using cotinine-confirmed smoking exposure assessment, especially for passive smoking, in areas of higher TB prevalence would help to more accurately determine the specific TB burden due to tobacco use. Comprehensive global tobacco control including active and passive smoking prevention and cessation efforts will likely further decrease the prevalence of LTBI in the United States, especially among adults. Tobacco cessation programs among TB patients as well as encouraging smoke-free homes for TB patients have already been recommended by The Union Against Tuberculosis and Lung Disease [43]. Furthermore, cigarette smoking exposure has been recommended as a criterion for treatment of LTBI [44]and has been incorporated into guidelines in the United States [45] and Canada [46]. Yet only Canada currently recommends smoking as criterion for TB testing among the already immigrated foreign-born. The US foreign-born who smoke or are exposed to smoke represent a subpopulation at particular risk for LTBI. Thus, the United States should also recommend TB testing for the already immigrated foreign-born that actively smoke or are exposed to passive smoke. Global tobacco control efforts should be joined with TB control programs in order to reduce morbidity and mortality associated with TB.
